# Changes in T Cell and Dendritic Cell Phenotype from Mid to Late Pregnancy Are Indicative of a Shift from Immune Tolerance to Immune Activation

**DOI:** 10.3389/fimmu.2017.01138

**Published:** 2017-09-15

**Authors:** Nishel Mohan Shah, Anna A. Herasimtschuk, Adriano Boasso, Adel Benlahrech, Dietmar Fuchs, Nesrina Imami, Mark R. Johnson

**Affiliations:** ^1^Department of Surgery and Cancer, Imperial College London, Chelsea and Westminster Hospital, London, United Kingdom; ^2^Department of Medicine, Imperial College London, Chelsea and Westminster Hospital, London, United Kingdom; ^3^Medical Research Council Human Immunology Unit, Weatherall Institute of Molecular Medicine and Nuffield Department of Medicine, University of Oxford, Oxford, United Kingdom; ^4^Division of Biological Chemistry, Biocenter, Innsbruck Medical University, Innsbruck, Austria

**Keywords:** pregnancy, immune modulation, immune response, tolerance, activation

## Abstract

During pregnancy, the mother allows the immunologically distinct fetoplacental unit to develop and grow. Opinions are divided as to whether this represents a state of fetal-specific tolerance or of a generalized suppression of the maternal immune system. We hypothesized that antigen-specific T cell responses are modulated by an inhibitory T cell phenotype and modified dendritic cell (DC) phenotype in a gestation-dependent manner. We analyzed changes in surface markers of peripheral blood T cells, *ex vivo* antigen-specific T cell responses, indoleamine 2,3-dioxygenase (IDO) activity (kynurenine/tryptophan ratio, KTR), plasma neopterin concentration, and the *in vitro* expression of progesterone-induced blocking factor (PIBF) in response to peripheral blood mononuclear cell culture with progesterone. We found that mid gestation is characterized by reduced antigen-specific T cell responses associated with (1) predominance of effector memory over other T cell subsets; (2) upregulation of inhibitory markers (programmed death ligand 1); (3) heightened response to progesterone (PIBF); and (4) reduced proportions of myeloid DC and concurrent IDO activity (KTR). Conversely, antigen-specific T cell responses normalized in late pregnancy and were associated with increased markers of T cell activation (CD38, neopterin). However, these changes occur with a simultaneous upregulation of immune suppressive mechanisms including apoptosis (CD95), coinhibition (TIM-3), and immune regulation (IL-10) through the course of pregnancy. Together, our data suggest that immune tolerance dominates in the second trimester and that it is gradually reversed in the third trimester in association with immune activation as the end of pregnancy approaches.

## Introduction

Changes occurring in the maternal immune system during pregnancy allow the semiallograft fetoplacental unit to develop and grow. To a degree HLA discordance assists fetal development and reduces the risk of vertical virus transmission such as HIV-1 (1). However, tight immunomodulation is required at the maternal–fetal interface to prevent fetal rejection. The observation that some immune disorders such as rheumatoid arthritis and multiple sclerosis typically improve during pregnancy supports the idea that pregnancy is associated with a state of immune tolerance (2–4). However, not all aspects of the immune system are downregulated, for example, innate immune responses are enhanced and have been associated with preterm labor. Conversely, regulatory T cell (Treg) responses are suppressed, and pro-inflammatory T cell activity is increased in conditions such as pre-eclampsia (5–7).

Various regulatory mechanisms repress maternal immune function and the response against fetal antigens, in particular at the maternal–fetal interface. Programmed death-1 (PD-1) expression on T cells may promote apoptosis of T cells specific for paternal antigens (8, 9). Furthermore, progesterone-regulated HLA-G expression on trophoblast initiates Fas/Fas-ligand (CD95/CD95L)-mediated apoptosis of maternal CD8^+^ T cells specific for paternal antigens (10).

Regulatory T cells have been suggested to modulate the function of the maternal immune system during pregnancy by suppressing effector T cell function (11). For example, Tregs promote effector T cell anergy and inhibit proliferation and cytokine secretion by CD4^+^CD25^−^ T cells under polyclonal stimulus (12), independent of antigen specificity (13). Tregs also mediate immune suppression by reducing the expression of costimulatory molecules CD80 and CD86 on antigen-presenting cells (APCs), and stimulating indoleamine 2,3-dioxygenase (IDO) enzymatic activity in dendritic cells (DCs) and macrophages (13–15). IDO is an enzyme that catalyzes the rate limiting step of the breakdown of the essential amino acid tryptophan (trp) into the kynurenine (kyn) pathway. The combination of trp depletion and accumulation of downstream bioproducts of kyn exert inhibitory activity on T cells *in vitro* (16), IDO inhibition *in vivo* causes immune-mediated fetal rejection in semiallogeneic but not syngeneic pregnancies in mice (17). Pregnancy appears to promote immature/tolerant DCs and suppresses their pro-inflammatory cytokine responses (18). Under steady state conditions, with abundant antigenic stimulation, the majority of peripheral DCs display an immature phenotype (19). When matured under the influence of IL-10, immature DCs develop a tolerant DC (DC^IL-10^) phenotype. These DCs secrete reduced quantities of pro-inflammatory cytokines and express low levels of MHC class 1 and 2 and costimulatory molecules (19). Tolerogenic DCs classically encourage CD4^+^ T cell differentiation into Treg and T-helper 2 (Th2) subtypes (20).

The majority of DCs are IFN-γ producing plasmacytoid (pDC) and non-lymphoid conventional or myeloid DC (cDC/mDC) (21, 22). Typically, pDC secretes large quantities of IFN-γ in response to virus and prime cytotoxic T cells against viral antigens, whereas mDC maintains self-tolerance and induces specific immune responses to foreign pathogens (23). In pregnancy, proportions of mDC and pDC have been shown to fall in the second trimester but subsequently increase in late pregnancy (24–26). The ratio of mDC and pDC shows a similar increase that is consistent with a predominance of mDCs. In addition, these DC populations become more activated during pregnancy, expressing increasing proportions of costimulatory markers and inflammatory cytokines (24, 26). Neopterin is an established biomarker of immune activation which is a product of guanosine-5′-triphosphate (GTP) catabolism in monocytes (27). Type I interferons are potent inducers of neopterin from activated human monocyte-derived macrophages and DCs (27, 28). Unsurprisingly, neopterin concentrations have been shown to increase with gestation in pregnancy, reflecting increasing DC and monocyte activation (29, 30).

Progesterone is important for the establishment and maintenance of pregnancy and exerts immune-modulatory effects mediated by the lymphocyte-derived protein progesterone-induced blocking factor (PIBF) (31, 32). Effector functions modulated by PIBF include cytokine synthesis, cytotoxic cell activity, and arachidonic acid synthesis (6, 31, 33). PIBF is produced by activated T cell receptor (TCR)-γδ^+^ and CD56^+^ T cells that have interacted with trophoblast and express progesterone receptors (PRs) (34). Serum concentrations of progesterone increase throughout pregnancy and can reach 175–636 nmol/l in maternal circulation mid third trimester (35). However, compared to the maternal–fetal interface, progesterone concentration peripherally is lower in humans and so systemically its immunemodulatory effects may be determined by lymphocyte sensitivity to the hormone (31).

Despite the influence of immune modulation, the expression of activation markers is increased on circulating and decidual T cells in late pregnancy and perhaps prior to the onset of labor (36, 37). This may suggest greater maternal immune awareness of the conceptus and trigger effector responses that could be involved in the process of parturition. In mice, this has been proposed as a cause of immune mediated fetal demise in fetal intervention (38).

In this study, we analyzed the *in vitro* functional responses to common recall antigens, profiled the changes in markers of peripheral blood T cell activation and maturation associated with pregnancy, and the *in vitro* expression of PIBF on lymphocytes in response to progesterone. Our results describe for the first time the course of pregnancy as a transition from downregulation to reconstitution of immune responses associated with modulation of T-cell and APC phenotype and function.

## Materials and Methods

### Ethics Statement

The Research Ethics Committee, Chelsea and Westminster Hospital Trust approved this study; Ref: 11/LO/0971. Human experimentation guidelines of the author’s institution were followed in the conduct of clinical research. Informed written consent was obtained from each donor prior to blood collection.

### Study Participants

For enzyme-linked immunosorbent spot (ELISpot) assays, blood was taken from 15 healthy volunteers (median 25.6 years, interquartile range, IQR 24.5–30.2 years), and 10 pregnant (Pr) (median 33.6 years, IQR 28.0–36.8 years) longitudinally in their second trimester (median 20.0 weeks, IQR 19.3–24.4 weeks) and in their third trimester (median 38.4 weeks, IQR 28.0–38.9 weeks). For peripheral blood flow cytometry, neopterin and trp concentrations and trp metabolic analysis, samples were obtained from 20 healthy Pr women, of whom 10 were in their second trimester (12–28 weeks of gestation) and 10 were in their third trimester (greater than 28 weeks of gestation). Median gestations were 21.6 weeks (IQR, 19.1–23.0 weeks) and 39.0 weeks (IQR 38.0–40.0 weeks), respectively, and a median age for all Pr was 35.0 years (IQR 30.0–36.0 years). Nine healthy non-Pr control female subjects (healthy control, HC) had a median age of 31.0 years (IQR 27.8–38.0 years). For progesterone cell culture experiments, blood was obtained from nine Pr (median age 33.0 years, IQR 28.5–36.5 years) of whom four were in their second trimester (median gestation 24.4 weeks, IQR 22.4–27.4 weeks) and five were in their third trimester (median 32.6 weeks, IQR 30.3–39.4 weeks), and five HC (median age 30.0 years, IQR 23.5–34.8 years).

### Blood Collection and Separation

Blood was collected in lithium-heparin-treated vacutainers (Becton Dickinson, Oxford, UK). Peripheral blood mononuclear cells (PBMCs) were isolated within 2 h of blood collection by a standard density gradient centrifugation with lymphocyte separation medium (PAA, Yeovil, UK), as previously described (39). PBMCs were counted using trypan blue exclusion, and viability was >80%.

### Measurement of IFN-γ, IL-10, Granzyme B, and IL-4 Production

IFN-γ, IL-10, granzyme B, and IL-4 ELISpot assays were used in order to quantify cellular responses following manufacturer recommendations (Mabtech, Stockholm, Sweden) and as previously described (39–41). Briefly, 1–2 × 10^5^ PBMCs per well were cultured in duplicate in media consisting of RPMI 1640 (Sigma) supplemented with penicillin, streptomycin, l-glutamine (final concentration 100 IU/ml, 100 µg/ml, and 2 mM, respectively), and 10% human male heat inactivated serum (Sigma), in 96-well polyvinylidene difluoride-backed plates (Millipore, Watford, United Kingdom) coated with anti-IFN-γ, anti-IL-10, anti-granzyme B, or anti-IL-4 monoclonal antibodies (Mabtech). Cells were stimulated with antigens or peptides at 5 µg/ml or phytohemagglutinin (Sigma) (positive control) at 5 µg/ml in culture medium. Antigens/peptides used included CMV, EBV, HSV, and influenza A whole lysates (Virion, Würzburg, Germany), measles, tetanus toxoid (TTOX), purified protein derivative (PPD), and flu, EBV and CMV peptide pool (FEC) (NIBSC, Potters Bar, UK). Negative controls comprised of cells cultured in media alone. IFN-γ-, IL-10-, and granzyme B-coated plates were incubated overnight at 37°C and 5% CO_2_ for 48 h and IL-4-coated plates for 96 h. Spot-forming cells (SFC) were then detected according to the manufacturer’s instructions (Mabtech). A positive result is defined as a score of 20 or more SFC per 10^6^ PBMC and at least three-fold greater than the negative control wells.

### Progesterone Cell Culture

Isolated PBMCs were cultured in six-well flat bottom culture plates (Sigma-Aldrich, Poole, UK) at a concentration of 1 × 10^6^ cells/ml in a final volume of 3 ml per well (3 × 10^6^ cells in total) and incubated for 24 h in 5% CO_2_ at 37°C. Culture medium consisted of RPMI 1640 (PAA) supplemented with 100 IU/ml penicillin, 100 µg/ml streptomycin (Sigma-Aldrich), 2 mM l-glutamine (PAA), 10% sterile filtered, and heat-inactivated male AB serum (Sigma-Aldrich), and either no progesterone (control), 0.64, 6.4, or 64 mM progesterone (Sigma-Aldrich). Cells were recounted and viability assessed (>80%) prior to flow-cytometric analysis.

### Flow Cytometry

Six-color flow cytometry was used to phenotype CD4 and CD8 T cell subsets. Cells were stained with the following murine anti-human monoclonal antibodies according to the manufacturer’s instructions: V500 labeled anti-CD127; peridinin chlorophyll protein (PerCP) Cy5.5-labeled anti-CD3; allophycocyanin (APC)-H7-conjugated anti-CD8; fluorescein isothiocyanate (FITC)-labeled anti-CD57, anti-programmed death-1 (PD-1; CD279), and anti-CD27; phycoerythrin (PE)-conjugated anti-CD38, anti-CD25; APC-labeled anti-CD95, anti-CD45RA, and anti-CD28; PECy7-labeled anti-HLA-DR, anti-PD-ligand 1 (PD-L1; CD274), and anti-CD45RO (all BD Biosciences, Oxford, UK); and PE-conjugated anti-CCR7 (R&D Systems, Abingdon, UK). Approximately 2 × 10^6^ cells per tube were stained, incubated in the dark at room temperature for 30 min, washed with PBS and fixed with BD stabilizing fixative (BD Biosciences), before acquisition within 24 h. At least 100,000 live events, gated on lymphocytes, were acquired on a 3-laser flow cytometer (BD Biosciences LSR II). Subsequently lymphocyte populations were gated according to respective isotype controls (Figure S1A in Supplementary Material).

In the same manner, flow cytometry was used to identify DCs of mDC and pDC lineage, as well as the expression of the costimulatory marker CD86. Antibodies used were as follows: FITC-labeled anti-Lin-1 (containing CD3, CD14, CD16, CD19, CD20, and CD56) (BD), PE-labeled anti-CD123 (BD), APC-labeled anti-CD86 (Biolegend, London, UK), PE-Cy7-conjugated anti-CD14 (BD), PerCP-5.5-conjugated anti-HLA-DR (eBiosciences, Hatfield, UK), and APC-H7-labeled anti-CD8 (BD). A minimum of 500,000 live events, gated on live cells, was acquired according to the description detailed above (Figure S1B in Supplementary Material).

Phenotyping following progesterone cell culture was performed to determine expression of PIBF and TCR-γδ on lymphocytes using antibodies: anti-CD3, anti-CD8, TCR-γδ (BD Biosciences), and rabbit anti-PIBF (Biorbyt, Cambridge, UK). A minimum of 200,000 events was acquired according to the description detailed above. Analysis of flow cytometric data was performed using FlowJo version 7.65 (Tree Star Inc, Ashland, OR, USA).

### Neopterin and IDO Activity

In a representative sample of 4 s and six third trimester pregnancies neopterin levels were determined by ELISA (ELItest Neopterin, BRAHMS Diagnostica, Henningsdorf, Germany). Kyn and Trp were measured in cell culture supernatants by HPLC (42); the Kyn/Trp ratio was calculated as an estimate of IDO activity.

### Statistical Analysis

All statistical analyses were performed using non-parametric tests as sample distribution cannot be assumed to follow a Gaussian profile. Data are expressed as median and IQR. Longitudinal comparison between second and third trimesters was analyzed in ELISpot responses. These data were analyzed using Wilcoxon matched-pairs signed ranks test. All other comparisons were cross-sectional and therefore Mann–Whitney *U* test was used to compare between group averages. Kruskal–Wallis one-way analysis of variance was used for multiple groups, and where appropriate Dunn’s test was used to adjust *p*-values for multiple group comparisons. Details are given in figure legends. Statistical analysis was undertaken using GraphPad Prism 6 (GraphPad Software, San Diego, CA, USA). All *p*-values presented are two sided, and *p* < 0.05 were considered significant.

## Results

### *In Vitro* Responses to Recall Antigens Are Altered during Gestation

**Figure 1:**
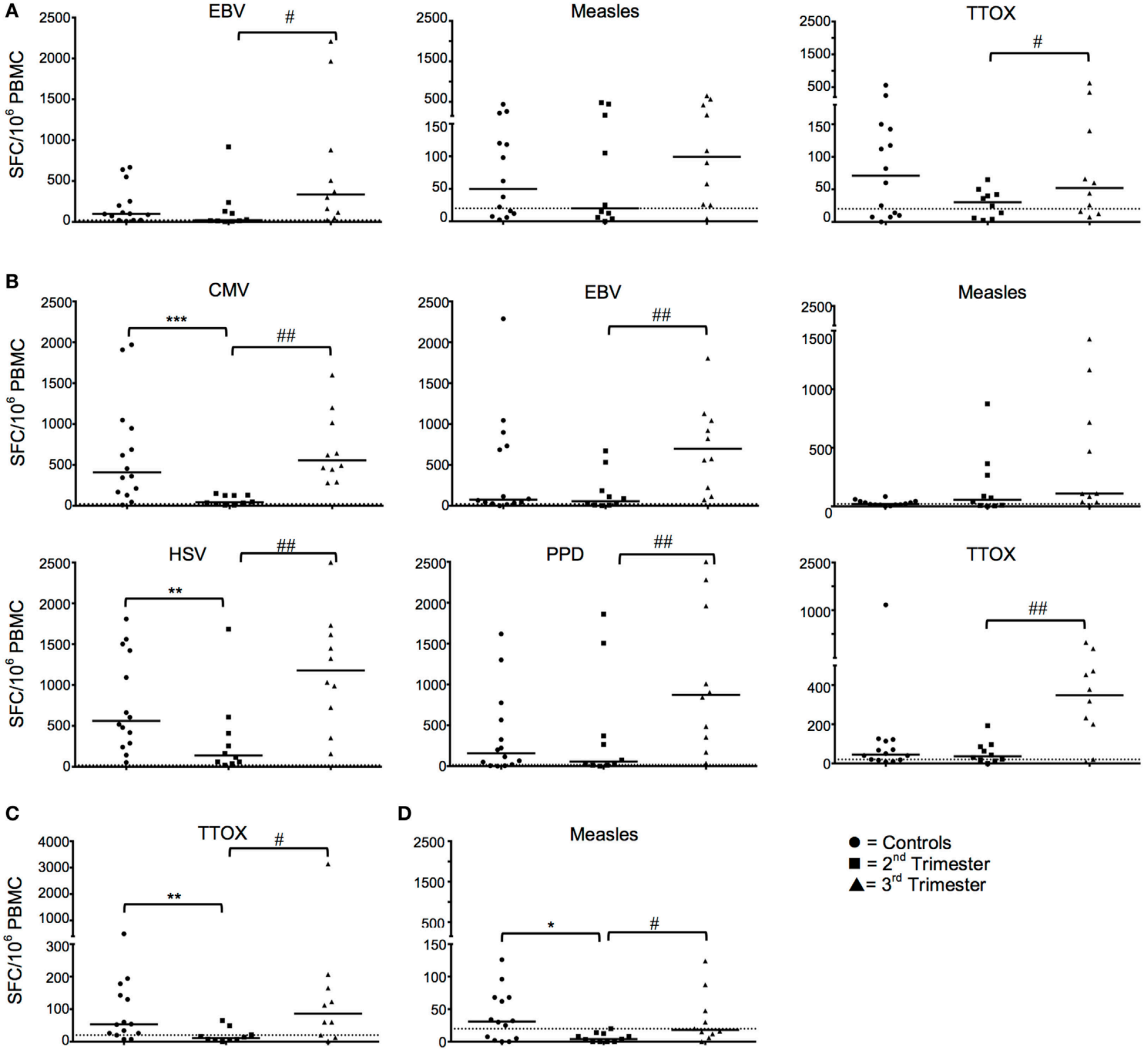
Longitudinal functional responses to recall antigens and peptides show dampened IFN-γ and IL-10 in the second trimester that revert in the third. **(A)** Shows numbers of IFN-γ producing cells in pregnancy in response to EBV, measles, and tetanus toxoid (TTOX), compared to healthy controls. **(B)** Likewise, panel B shows pregnancy trimester changes of IL-10 cellular responses to CMV, EBV, HSV, purified protein derivative (PPD), and TTOX, compared to non-pregnant controls. **(C,D)** Granzyme and IL-4 responses to TTOX and measles, respectively. ^♯^*p* < 0.05, ^♯♯^*p* < 0.01 (Wilcoxon matched-pairs signed ranks test); *p < 0.05, **p < 0.01, ***p = 0.001 (Mann–Whitney-*U*). Abbreviation: PBMC, peripheral blood mononuclear cell.

IFN-γ ELISpot responses against EBV and TTOX (Figure 1A), and IL-10 responses against CMV, EBV, measles, HSV, PPD, and TTOX (all *p* < 0.05; Figure 1B) were all reduced in the second trimester. IFN-γ recall antigen responses returned to levels comparable to HC in the third trimester (Figure 1A). However, IL-10 responses against EBV, PPD, Measles, HSV, and TTOX in the third trimester were greater than HC. Numbers of IL-4- and granzyme B-producing cells in response to the aforementioned antigens and peptides were predominantly stable across gestation. However, granzyme B responses against TTOX were downregulated in the second trimester (Figure 1C), and a similar trend was observed in responses against measles (*p* = 0.0525) (data not shown). Cellular IL-4 production against measles showed a gestational increase but was also significantly reduced in second trimester pregnancies compared to healthy controls (*p* = 0.0194, Figure 1D).

### Pregnancy Is Associated with a Shift in T Cell Maturation toward an Effector Memory Subtype

**Figure 2:**
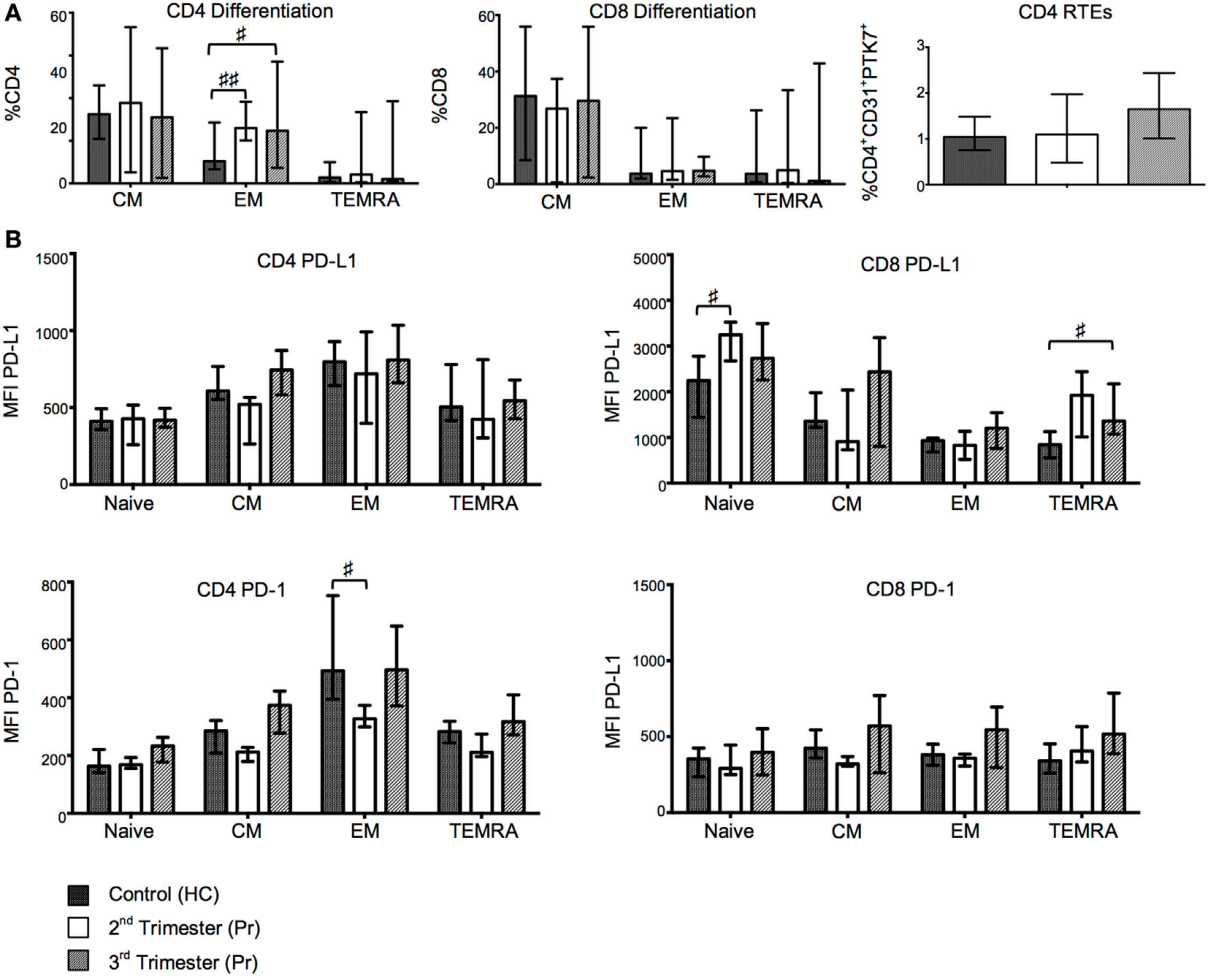
Changes in T-cell maturation and exhaustion with normal pregnancy. **(A)** Shows the frequencies of naïve, EM, CM, and TEMRA CD4 and CD8 T cells as determined by flow cytometry in pregnant (Pr) and health control (HC). Additionally, we show proportions of CD4 recent thymic emigrants determined by CD31 and PTK7 coexpression. **(B)** Programmed death-1 (PD-1) and PD ligand 1 (PD-L1) expression measured by mean florescence intensity (MFI) on naïve, EM, CM, and TEMRA CD4 and CD8 T cells. Columns indicate median and error bars interquartile range. ^♯^*p* < 0.05 and ^♯♯^*p* < 0.01 (Kruskal–Wallis one way analysis of variance and Dunn’s test for multiple comparisons).

Central memory (T_cm_/CM), effector memory (T_em_/EM), naïve, and terminally differentiated effector memory (T_Temra_/TEMRA) T cell subsets were defined using the surface markers CCR7 and CD45RA. The drive toward CD4 EM T cell subset is marked in the second and third trimester where the frequency of EM CD4 T cells was significantly higher than that observed in HC (Figure 2A). No statistically significant differences were observed in the CD8^+^ T cell subpopulation (Figure 2A).

### Central Naive T Cells and Recent Thymic Emigrants (RTE) Remain Constant with On-Going Pregnancy

The frequency of PTK7 expressing RTE CD4 T cells was comparable between Pr and HC (Figure 2A). In addition, we did not observe any difference in percentages of central naive CD4^+^ nor CD8^+^ T cells (defined as CD31^−^CD45RA^+^). Similarly, T cell differentiation, defined by CD27 and CD28 coexpression on CD4^+^ and CD8^+^ T cell populations, was unaltered in pregnancy (data not shown).

### Gestation Is Associated with Increases in the Expression of Exhaustion Markers

Programmed death-1 and PD-L1 expression was measured in CM, EM, naïve, and TEMRA CD4^+^ and CD8^+^ T cell subsets. The mean florescence intensity (MFI) of PD-1 on EM CD4 T cells showed gestational variation with a significant reduction in Pr compared to HC in the second trimester (*p* = 0.0219). Cell-surface expression of PD-L1 was increased in both the TEMRA and naive CD8^+^ T cell subsets and primarily occurred during the second trimester (Figure 2B). No significant differences were found in the expression of PD-L1 on CD4^+^ T cells or PD-1 on CD8 T cells (Figure 2B).

### Advancing Pregnancy Is Associated with Upregulation of T Cell Activation Markers

**Figure 3:**
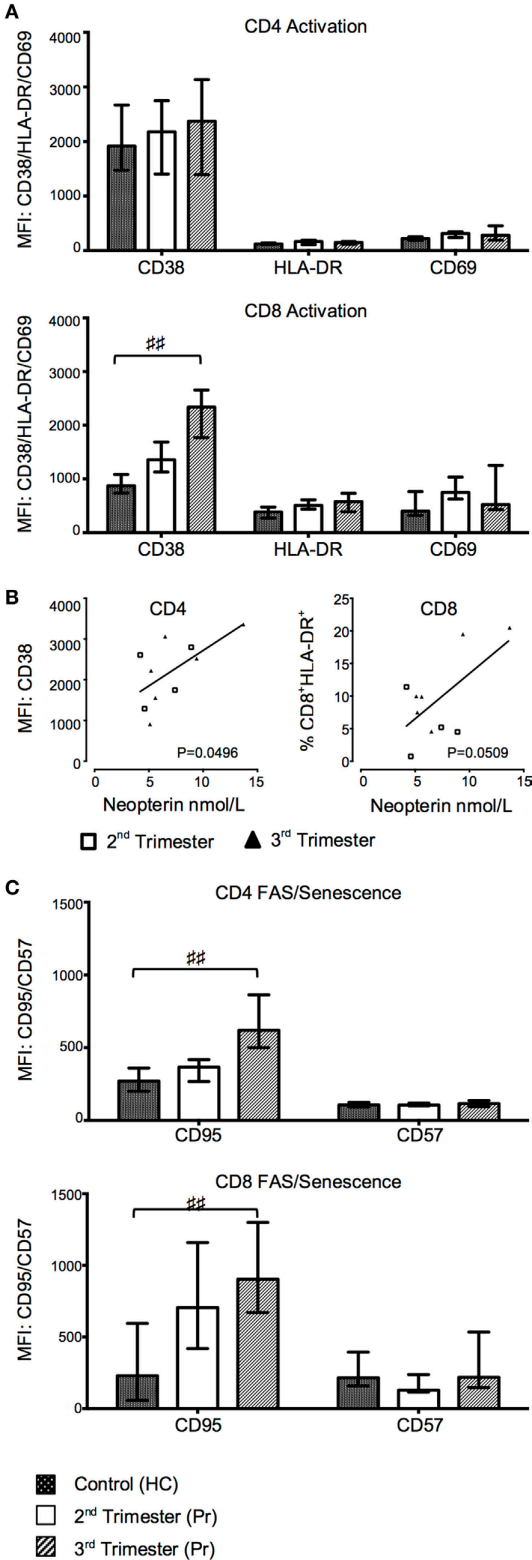
Gradual increase in T cell activation and pro-apoptotic marker expression during normal pregnancy. **(A)** Changes in activation markers CD38, HLA-DR, and CD69 expression in CD4 and CD8 T cells with trimesters and compared to controls. **(B)** Plasma neopterin concentration in pregnancy correlated with CD38 mean florescence intensity (MFI) on CD4^+^ T cells. **(C)** FAS and CD57 marker expression on CD4 and CD8 T cells during pregnancy and compared to health controls (HCs). Columns indicate median and error bars interquartile range. ^♯♯^*p* < 0.01 (Kruskal–Wallis one way analysis of variance and Dunn’s test for multiple comparisons). Abbreviation: Pr, pregnant.

CD38 MFI and frequency of CD38 progressively increased in CD8^+^ T cells from Pr in relation to the gestation period and were significantly higher in the third trimester Pr compared with HC (CD38 MFI *p* = 0.0111; %CD38^+^CD8^+^ T cells *p* = 0.0419; Figure 3A). There were no statistically significant differences between the two groups in the expression of activation markers CD69 and HLA-DR on CD8 and CD4 T cells (Figure 3A). However, plasma neopterin concentration in pregnancy positively correlated with CD38 MFI on CD4^+^ T cells (*p* = 0.0496; Figure 3B). A similar trend was seen in relation to CD8^+^HLA-DR^+^ T cells (*p* = 0.0509).

### Third Trimester of Pregnancy Is Accompanied by Greater Expression of T Cell Pro-Apoptotic Markers

We measured the expression of the pro-apoptotic receptor CD95 and the senescence marker CD57 on CD4 and CD8 T cells. The MFI of CD95 on both CD4 (*p* = 0.0084) and CD8 (*p* = 0.0077) T cells was also significantly increased in third trimester of pregnancy as compared to HC (Figure 3C). Consequently, the CD57^−^CD95^+^ subset was increased in the CD4^+^ T cell compartment in Pr compared to HC (*p* = 0.0060; data not shown) and was accompanied by a corresponding decrease in CD57^−^CD95^−^ CD4 T cells. This difference was primarily seen in the third trimester (*p* = 0.0074). There was no difference in the cell-surface density of CD57 between groups (Figure 3C).

### Proportions of Regulatory T-Cell Subsets Are Increased with Advancing Pregnancy

**Figure 4:**
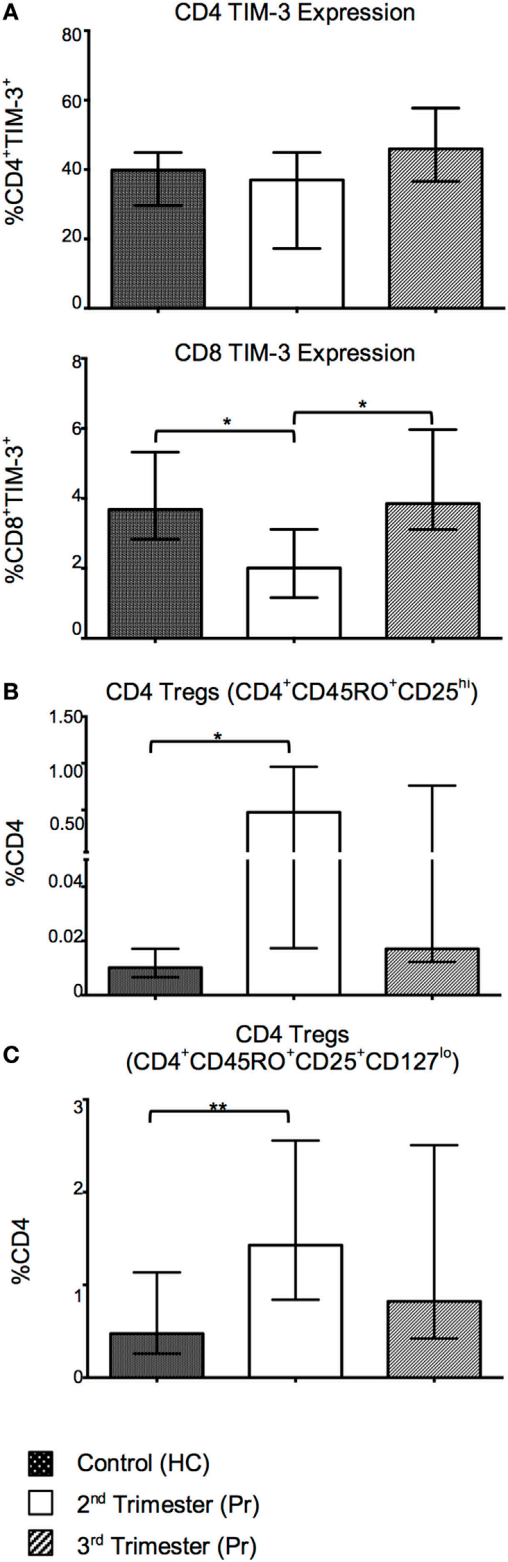
Tolerant T-cell phenotypes are favored in pregnancy and reflect changes in T-cell activation with increasing gestation. **(A)** Percentage expression of CTLA-4 on TIM-3 negative and positive, CD4 and CD8 T cells in pregnancy compared with controls. **(B,C)** Proportions of CD4 regulatory T cells (Tregs) in pregnancy. These cells were identified using the phenotype CD4^+^CD45RO^+^CD25^hi^ and CD4^+^CD45RO^+^CD25^+^CD127^lo^. *p < 0.05 and **p < 0.01. Columns indicate median and error bars interquartile range. *p < 0.05 and **p < 0.01 (Mann–Whitney *U*). Abbreviation: HC, health control; Pr, pregnant.

With the aim of investigating alternative T cell markers associated with immune regulation, we analyzed expression of inhibitory ligand CTLA-4 and negative regulatory marker TIM-3. The proportion of CD8 TIM-3^+^ T cells was reduced in the second trimester and returned to normal in the third (Figure 4A). CD4 TIM-3 showed no gestation variation. Surface CD4 and CD8 CTLA-4 expression was minimal in our cohort (data not shown). There was also a significant increase in the proportion of Tregs, defined as CD4^+^CD45RO^+^CD25^hi^ and CD4^+^CD45RO^+^CD25^+^ CD127^lo^ in Pr samples in the second trimester when compared to controls (Figures 4B,C).

### Proportions of mDC and Costimulatory CD86 Expression on pDC Were Reduced in Pregnancy

**Figure 5:**
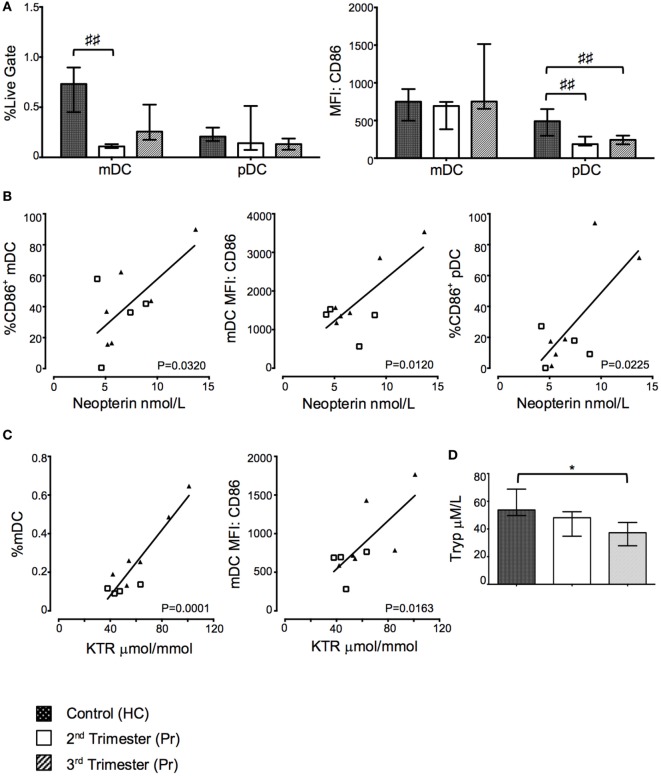
Proportions of myeloid dendritic cell (mDC) and costimulatory CD86 expression on plasmacytoid DC (pDC) were reduced in pregnancy but positively correlated with indoleamine 2,3-dioxygenase (IDO) activity. **(A)** Proportions of mDC and pDC and their respective expression of CD86 in pregnancy and compared to controls. **(B)** Correlation between CD86 expression on DCs and plasma neopterin concentration. **(C)** Correlation between mDC proportions, CD86 expression, and plasma kynurenine/tryptophan ratio (KTR). **(D)** Plasma tryptophan, kyurenine concentration, and KTR (μmol/mmol) during pregnancy. Columns indicate median and error bars interquartile range. ^♯♯^*p* < 0.01 (Kruskal–Wallis one way analysis of variance and Dunn’s test for multiple comparisons), *p < 0.05 (Mann–Whitney *U*). Abbreviations: HC, healthy control; MFI, mean florescence intensity; Pr, pregnant.

Myeloid DC and pDC were defined as CD14^−^Lin^−^HLA-DR^hi^CD123^−^ and CD14^−^Lin^−^HLA-DR^+^CD123^+^, respectively. The percentage of mDC, but not pDC was significantly reduced in Pr samples compared to HC (*p* = 0.0016). This difference was observed predominately during the second trimester (Figure 5A). We observed reduced CD86 MFI on pDC with gestation when compared to HC (*p* = 0.0038; Figure 5A).

### Greater Proportion of Activated mDC But Not Tregs Correlates with Increased Kynurenine/Tryptophan Ratio (KTR)

Plasma neopterin concentration positively correlated with greater proportions of CD86 expressing mDC (*p* = 0.0320; Figure 5B) and pDC (*p* = 0.0225; Figure 5B) as well as CD86 MFI on mDC (*p* = 0.0120; Figure 5B). Furthermore, KTR positively correlated with increasing proportions of mDC (*p* = 0.0001; Figure 5C) and CD86 expression on mDC (*p* = 0.0163; Figure 5C). Interestingly, these changes appear to be driven by third trimester pregnancies as depicted on the *x*–*y* scatter graphs. There was no statistically significant relationship between Treg proportions and trp and neopterin concentrations or KTR (data not shown). The changes in KTR appeared to be driven primarily by a reduction in trp concentration through gestation (*p* = 0.0256; Figure 5D), whereas increases in kyn were not observed in Pr compared to HC (Figure 5D).

### Proportions of CD4^+^ and CD8^+^ TCR-γδ^+^ T Cells Are Reduced during Pregnancy But CD8^+^TCR-γδ^+^ T Cells Express Greater PIBF

**Figure 6:**
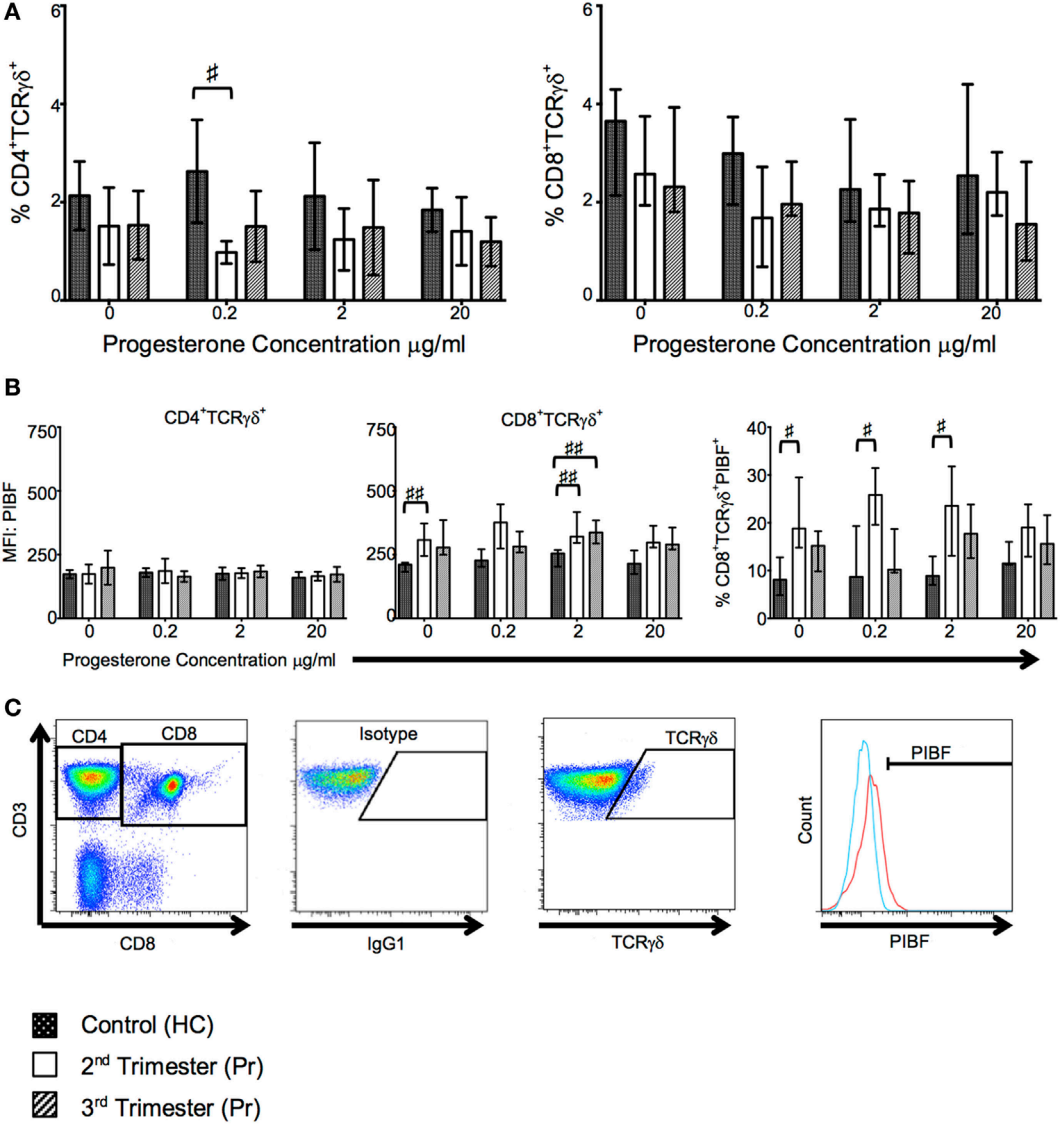
T cell receptor (TCR)-γδ^+^ T-cell proportions are reduced in pregnancy but they express greater progesterone-induced blocking factor (PIBF). These changes are primarily seen in the second trimester. **(A)** Shows changes in proportions of TCR-γδ CD4 and CD8 T cells in response to progesterone culture. **(B)** Alterations in PIBF expression on the aforementioned TCR-γδ T cells. **(C)** Peripheral blood mononuclear cell (PBMCs) were surface stained with anti-CD3, anti-CD8, anti-TCR-γδ, and anti-PIBF. Gating strategy used to phenotype CD4^+^/CD8^+^ TCR-γδ^+^ PIBF^+^ T-cells. Box and whiskers plots indicate median, interquartile range and 10th–90th centiles. ^♯^*p* < 0.05 and ^♯♯^*p* < 0.01 (Kruskal–Wallis one way analysis of variance and Dunn’s test for multiple comparisons). Abbreviations: HC, healthy control; Pr, pregnant.

In our cell culture experiments, we found that proportions of CD4^+^TCR-γδ^+^ but not CD8^+^TCR-γδ^+^ T cells were reduced in Pr compared with HC following culture with low concentrations of progesterone; interestingly this reduction was observed in the second trimester only (*p* = 0.0120, Figure 6A). This difference was mitigated and lost statistical significance when higher concentrations of progesterone were used. Additionally, PIBF expression on CD8^+^TCR-γδ^+^ T cells was significantly increased during pregnancy independent of progesterone stimulation, a phenomenon which seemed to be predominant in second trimester pregnancies as suggested by the percentage of PIBF expressing CD8^+^TCR-γδ^+^ T cells (Figure 6B). No changes in PIBF expression were observed when CD4^+^ T cells were analyzed (Figure 6B). Overall, there seem to be no differences in PIBF expressing cells between increasing progesterone concentrations used during *in vitro* culture. This may suggest a loss of sensitivity to progesterone in the second and third trimester due to over saturation or pregnancy related immune modulation. The gating strategy used to analyze TCR-γδ and PIBF expression is shown in Figure 6C.

## Discussion

Our studies of immune parameters in normal pregnancy describe immune quiescence and inhibition of responses in the second trimester, which reverse in the third trimester in association with greater immune activation and functional immune responses. We suggest that a complex temporal relationship between activation and exhaustion determines the functional status of the maternal immune system during pregnancy.

Previous studies have described gestational increases in plasma IFN-γ and IL-10, and others have shown comparably greater levels of IL-10 and reduced IFN-γ production, measured by multiplex assays, in response to LPS in pregnancy than that observed in unstimulated PBMC suggesting a tendency toward immune repression (43–46). Here, we take a different approach, assessing cytokine responses by T cells following recall antigen stimulation, which are largely mediated by EM and TEMRA subsets (47). Other studies have shown that IL-10 ELISpot PBMC responses are increased in pregnancy (48) and IFN-γ and IL-10 decidual MNC responses are increased in labor (49). The effect of pregnancy may be to negatively affect pleiotropic pro-inflammatory cytokine production and cause immune cells to produce comparably greater IL-10 than in the non-Pr individual (44, 45). Furthermore, in some infective pathology, outside of pregnancy, IL-10 serves to suppress antigen specific (ELISpot) T cell responses *ex vivo* (50). Our results show that IFN-γ responses are reduced in the second trimester compared to third trimester pregnancies, despite the observed increase in CD4 EM proportion in the second trimester, which have rapid effector functions and homing to inflamed tissues (47). This decline in responses reverses in the third trimester. This observation suggests that effective inhibition of CD4 responses characterizes the second trimester. IL-10 responses showed a pattern similar to IFN-γ, arguing against a role for antigen-induced IL-10 in immune tolerance in mid-gestation. However, IL-10 production may have a key role in the third trimester, holding the ascendant immune activation in check. Of note, although IL-4 and granzyme B responses were largely undetectable, they showed a similar pattern to IFN-γ and IL-10 when measurable. Thus, immune responses in the second trimester are largely muted but are subsequently restored to a non-Pr level in the third trimester. Therefore, we propose that immune tolerance predominates during the second trimester, which is reversed in the third trimester, requiring active immune suppression to prevent exaggerated immune responses. In this study, we sought to understand the potential mechanisms at play.

Programmed death-1 and PD-L1 exhaustion markers are expressed on not only activated lymphocytes and Tregs but also non-hematopoietic and non-lymphoid cells such as placenta (8). The interaction between PD-1 and its ligand can either negatively or positively affect leukocyte activation (8). PD-L1 blockade in mice leads to impaired FoxP3 induction (51), a reduction in Treg proportion and favors a shift toward Th17 T cell phenotype that represses feto-maternal tolerance, resulting in embryo reabsorption (52, 53). Therefore, in the context of pregnancy, PD-1 signaling is required for both the suppression of effector T cells and the maintenance and induction of Tregs. In addition, the PD-1-PD-L1 pathway may induce fetal-specific T cells apoptosis thus limiting their accumulation (8). Our observations suggest that during the second trimester, a selective dampening of primary CD8 T cell immune responses and CD4 EM immune quiescence occurs, which reverts in the third trimester. In addition, we found that Treg proportions were increased in the second trimester compared to controls in keeping with previous reports (54). However, this may not be an accurate reflection of their functional status.

Pregnancy is characterized by significant changes in endocrine hormones and receptors. A close relationship between the endocrine and immune system has previously been reported (55). It is likely that the majority of changes seen in pregnancy are driven by the hormones estrogen and progesterone (56–58). Increasing concentrations of both hormones positively correlate with increasing gestation and successful pregnancy (56, 59, 60). Estrogen has been shown to promote immune tolerance by inhibiting CD4 T cell expansion and promoting apoptosis, increasing Treg proportions, and promoting tolerant DC expressing inhibitory markers (61–63). In addition, T cells express other steroid hormone receptors including glucocorticoid (GR) and androgen (AR) receptors that enable modulation of T cell functions (64, 65). For example, progesterone can bind to GR on T cells to increase Treg proportions, and testosterone binds to AR to regulate Th1 differentiation and promote FoxP3 expressing Tregs (64–66), although the latter is less important in pregnancy.

Progesterone is important for the establishment and maintenance of pregnancy and exerts immunemodulatory effects (67). Some of these are mediated by the lymphocyte-derived protein PIBF (31, 32). PIBF production is mediated through PRs in TCR-γδ^+^ and CD8^+^ T cells. Indeed, *in vitro* treatment of leukemic cell lines and CD3^+^ T cells with progesterone increases PIBF protein expression (68–71). PIBF binds a GPI-anchored receptor in a heterodimer with the α-domain of the IL-4 receptor, activating STAT6, inducing a Th2 cytokine bias, and suppressing lymphocyte cytotoxicity (33, 72). TCR-γδ T cells comprise >70% of decidual, but only 1–2% of peripheral, lymphocytes. They become progesterone-responsive after exposure to fetoplacental antigens stimulates PR expression (31). Uniquely, they combine adaptive and innate-like responses and are able to interact with non-classical HLA antigens such as those expressed by trophoblast (31, 73). Our results show a fall in proportion of CD4 TCR-γδ^+^ T cells, which may suggest TCR-γδ hypoexpression or apoptosis. However, we have also shown a greater PIBF expression in CD8^+^TCR-γδ^+^ T cells under the influence of progesterone in the second trimester. With greater regulation of responder CD8 T cells in mid trimester, as suggested by the differential expression of exhaustion markers in TEMRA subsets, it seems the second trimester is also characterized by progesterone regulation of MHC unrestricted CD8 TCR-γδ^+^ T cells. Unexpectedly, progesterone did not increase the expression of PIBF in the CD4^+^TCR-γδ subset. This may be due to an absence of antigenic stimulus, or indicative of negative regulation of systemic immune activation at this stage of pregnancy; our samples were taken between 22–27 weeks and 30–39 weeks of gestation (IQR). Indeed, a previous study that demonstrated a concentration-dependent increase in PIBF expression, measured by flow cytometry, and used blood taken at 16–24 weeks of gestation, whereas our study has used a much wider gestational range (71). Urinary PIBF falls in the third trimester despite increasing progesterone levels, perhaps explaining our results (74, 75). In order to further elucidate the impact of pregnancy on adaptive immunity we also analyzed DC phenotype and IDO activity.

Pregnancy-specific cytokines and progesterone have been shown to modify DC maturation and their ability to influence T cell responses by promoting IL-10 production and increasing the number of iTregs (20, 76, 77). Profiling of DC in our experiments showed decreased proportions of mDC but not pDC in peripheral blood, with dampened immune activating phenotypes. It remains unclear whether this loss of DC in the periphery is due to apoptosis or redistribution to other sites. Furthermore, we demonstrated a correlation between mDC proportions, pDC and mDC activation, and neopterin concentration as well as between mDC proportion and IDO activity. These changes are predominately seen in the third trimester and are closely related to T cell activation. Previous studies have shown a falling trp concentration but increasing KTR with advancing gestation suggesting increased IDO activity (78). We found that trp concentration but not KTR showed a gestational reduction, which was not mirrored by increased kyn concentration. An alternative explanation is trp and metabolic steroids can induce trp 2,3-dioxygenase activity in the liver to catabolize trp (79). Thus, the fall in trp concentration maybe due to altered trp bioavailability to favor fetal development rather than a reflection of greater IDO activity (80). However, we cannot exclude the possibility that DC phenotype and IDO activity share the same gestational variation as seen on T cells with a “Yin and Yang” relationship between immune activation and regulation. IDO activity has previously been shown to correlate with neopterin concentrations in pregnancy (17).

The reconstitution of immune responses in the third trimester, suggested by our ELISpot data, is accompanied by greater immune activation. It is already known that pregnancy is associated with increased immune activation, typified by the high proportions of CD38^+^HLA-DR^+^ T cells throughout pregnancy and up to 6 months after delivery (81). Our data show that CD38 expression on CD8^+^ T cells gradually increases with gestation, and HLA-DR expression by CD8 T cells correlated with increasing concentrations of neopterin, which is a surrogate marker for IFN-γ-associated immune activation (82, 83).

Apoptosis has also been proposed as a means of regulating immune responses in pregnancy to favor the semiallograft. Analysis of CD95 expression on T cells in our cohort showed that its expression was increased on both CD4 and CD8 T cells progressively with gestation and in parallel to increased immune activation. The CD95-CD95L pro-apoptotic pathway may potentiate maternal immune tolerance during normal pregnancy. Thus, the expression of CD95L by trophoblast may induce apoptosis of CD95 expressing activated cytotoxic maternal T cells at the maternal–fetal interface. Consistent with this, different CD95 gene polymorphisms have been shown to alter the risk of early pregnancy loss (84, 85). Moreover, the absence of CD95 expression is associated with a reduction in the deletion of fetus-specific T cells, but this does not result in an increased incidence of fetal reabsorption (86). The outcome of CD95 ligation on T cells may depend on the dose of the signal and the cytokine environment suggesting a more complex immunologic scenario than suggested above (87).

Another inhibitory marker that may influence immune responses, TIM-3, is expressed on the surface of terminally differentiated Th1 but not Th2 cells and its signaling downregulates Th1 and Th17 responses by inducing apoptosis (88). These effects are galectin-9 mediated, which is expressed on gastrointestinal epithelium, endothelium, and immune cells. TIM-3 may be hormone and cytokine regulated with increased expression in pregnancy (89, 90). Our data suggest a reduction in CD8^+^TIM-3^+^ T cells in the second trimester of pregnancy that reverts to normal in the third. This reflects similar changes seen in CD95 and CD38 expression.

The reversal of immune tolerance suggested by our data may reflect the processes occurring prior to the onset of parturition. Labor is an inflammatory process and may be driven by fetal antigen exposure and potent innate immune stimulus. Previous reports have shown gestational-specific changes in IFN-γ, TNF-α, IL-2, IL-3, IL-6, IL-10, IL-12, and GM-CSF (43, 44, 91, 92). Inflammation *per se* can lead to the release of mitochondrial DNA due to tissue necrosis (93). In addition, labor is associated with an increase in corticotrophin-releasing hormone mRNA found in syncytiotrophoblast membrane microparticles (94). Placental-derived mitochondrial DNA and syncytiotrophoblast membrane microparticles-induced monocyte cytokine secretion may contribute to the inflammatory processes occurring before and during parturition (93, 95). Other reports have shown that decidua contains differentiated EM cells poised to respond to antigen and that fetal-specific T cells respond to placental antigens in draining LNs (37, 96–98). Nancy and Erlebacher postulated whether the presentation of antigen at the maternal–fetal interface serves to reinforce the activation of placenta-specific T cells that were first exposed to antigen elsewhere (96). Therefore, parturition appears to involve an array of triggers for cell-mediated and innate responses in a poised and activated immune system.

Collectively, our data indicate that the adaptive immune system from mid to late pregnancy is increasingly activated, with simultaneous negative regulation of responder T cells, suggesting a delicate balance between immune responses and immune tolerance. However, whereas immune suppression dominates in the second trimester, this gradually reverses in the third trimester.

## Ethics Statement

The Research Ethics Committee, Chelsea and Westminster Hospital Trust approved this study; Ref: 11/LO/0971. Human experimentation guidelines of the author’s institution were followed in the conduct of clinical research. Informed written consent was obtained from each donor prior to blood collection. This work was supported by Borne.

## Author Contributions

NS, NI, and MJ had a substantial contribution to the conception and design of the project and its interpretation. NS, NI, MJ, AeB, and DF were responsible for the acquisition, analysis, and interpretation of the data, and NS, AH, AdB, NI, and MJ drafted the work. All authors contributed to revising of the manuscript and have approved the final version. All authors agreed to be accountable for all aspects of the work in ensuring that questions related to the accuracy or integrity of any part of the work are appropriately investigated and resolved.

## Conflict of Interest Statement

The authors declare that the research was conducted in the absence of any commercial or financial relationships that could be construed as a potential conflict of interest. The handling editor declared a shared affiliation, though no other collaboration, with one of the authors, AB.

## References

[1] Mackelprang RD John-Stewart G Carrington M Richardson B Rowland-Jones S Gao X Maternal HLA homozygosity and mother-child HLA concordance increase the risk of vertical transmission of HIV-1. J Infect Dis (2008) 197(8):1156–61. 10.1086/529528 18462163 PMC2689391

[2] Weetman AP. Immunity, thyroid function and pregnancy: molecular mechanisms. Nat Rev Endocrinol (2010) 6(6):311–8. 10.1038/nrendo.2010.46 20421883

[3] Hughes GC Choubey D. Modulation of autoimmune rheumatic diseases by oestrogen and progesterone. Nat Rev Rheumatol (2014) 10(12):740–51. 10.1038/nrrheum.2014.144 25155581

[4] Tsui A Lee MA Multiple sclerosis and pregnancy. Curr Opin Obstet Gynecol (2011) 23(6):435–9. 10.1097/GCO.0b013e32834cef8f 22011954

[5] Molvarec A Shiozaki A Ito M Toldi G Stenczer B Szarka A Increased prevalence of peripheral blood granulysin-producing cytotoxic T lymphocytes in preeclampsia. J Reprod Immunol (2011) 91(1–2):56–63. 10.1016/j.jri.2011.03.012 21763002

[6] Hudić I Fatusić Z Szekeres-Bartho J Balić D Polgar B Ljuca D Progesterone-induced blocking factor and cytokine profile in women with threatened pre-term delivery. Am J Reprod Immunol (2009) 61(5):330–7. 10.1111/j.1600-0897.2009.00699.x 19343831

[7] Toldi G Rigó J Jr Stenczer B Vásárhelyi B Molvarec A. Increased prevalence of IL-17-producing peripheral blood lymphocytes in pre-eclampsia. Am J Reprod Immunol (2011) 66(3):223–9. 10.1111/j.1600-0897.2011.00987.x 21306467

[8] Taglauer ES Yankee TM Petroff MG. Maternal PD-1 regulates accumulation of fetal antigen-specific CD8+ T cells in pregnancy. J Reprod Immunol (2009) 80(1–2):12–21. 10.1016/j.jri.2008.12.001 19368976 PMC2764286

[9] Topalian SL Drake CG Pardoll DM. Targeting the PD-1/B7-H1(PD-L1) pathway to activate anti-tumor immunity. Curr Opin Immunol (2012) 24:207–12. 10.1016/j.coi.2011.12.009 22236695 PMC3319479

[10] Fournel S Aguerre-Girr M Huc X Lenfant F Alam A Toubert A Cutting edge: soluble HLA-G1 triggers CD95/CD95 ligand-mediated apoptosis in activated CD8+ cells by interacting with CD8. J Immunol (2000) 164(12):6100–4. 10.4049/jimmunol.164.12.6100 10843658

[11] Peck A Mellins ED. Plasticity of T-cell phenotype and function: the T helper type 17 example. Immunology (2010) 129(2):147–53. 10.1111/j.1365-2567.2009.03189.x 19922424 PMC2814457

[12] Ernerudh J Berg G Mjosberg J. Regulatory T helper cells in pregnancy and their roles in systemic versus local immune tolerance. Am J Reprod Immunol (2011) 66(Suppl 1):31–43. 10.1111/j.1600-0897.2011.01049.x 21726336

[13] Sakaguchi S Yamaguchi T Nomura T Ono M. Regulatory T cells and immune tolerance. Cell (2008) 133(5):775–87. 10.1016/j.cell.2008.05.009 18510923

[14] Kushwah R Hu J Role of dendritic cells in the induction of regulatory T cells. Cell Biosci (2011) 1(1):20 10.1186/2045-3701-1-20 21711933 PMC3125210

[15] Saito S Shiozaki A Sasaki Y Nakashima A Shima T Ito M. Regulatory T cells and regulatory natural killer (NK) cells play important roles in feto-maternal tolerance. Semin Immunopathol (2007) 29(2):115–22. 10.1007/s00281-007-0067-2 17621697

[16] Terness P Kallikourdis M Betz AG Rabinovich GA Saito S Clark DA. Tolerance signaling molecules and pregnancy: IDO, galectins, and the renaissance of regulatory T cells. Am J Reprod Immunol (2007) 58(3):238–54. 10.1111/j.1600-0897.2007.00510.x 17681041

[17] Munn DH Zhou M Attwood JT Bondarev I Conway SJ Marshall B Prevention of allogeneic fetal rejection by tryptophan catabolism. Science (1998) 281(5380):1191–3. 10.1126/science.281.5380.1191 9712583

[18] Butts CL Bowers E Horn JC Shukair SA Belyavskaya E Tonelli L Inhibitory effects of progesterone differ in dendritic cells from female and male rodents. Gend Med (2008) 5(4):434–47. 10.1016/j.genm.2008.11.001 19108816 PMC2941400

[19] Frick JS Grunebach F Autenrieth IB. Immunomodulation by semi-mature dendritic cells: a novel role of toll-like receptors and interleukin-6. Int J Med Microbiol (2010) 300(1):19–24. 10.1016/j.ijmm.2009.08.010 19781988

[20] Blois SM Kammerer U Alba Soto C Tometten MC Shaikly V Barrientos G Dendritic cells: key to fetal tolerance? Biol Reprod (2007) 77(4):590–8. 10.1095/biolreprod.107.060632 17596562

[21] Merad M Sathe P Helft J Miller J Mortha A. The dendritic cell lineage: ontogeny and function of dendritic cells and their subsets in the steady state and the inflamed setting. Annu Rev Immunol (2013) 31:563–604. 10.1146/annurev-immunol-020711-074950 23516985 PMC3853342

[22] Malissen B Tamoutounour S Henri S The origins and functions of dendritic cells and macrophages in the skin. Nat Rev Immunol (2014) 14(6):417–28. 10.1038/nri3683 24854591

[23] Merad M Manz MG. Dendritic cell homeostasis. Blood (2009) 113(15):3418–27. 10.1182/blood-2008-12-180646 19176316 PMC2668851

[24] Della Bella S Giannelli S Cozzi V Signorelli V Cappelletti M Cetin I Incomplete activation of peripheral blood dendritic cells during healthy human pregnancy. Clin Exp Immunol (2011) 164(2):180–92. 10.1111/j.1365-2249.2011.04330.x 21352205 PMC3087910

[25] Ueda Y Hagihara M Okamoto A Higuchi A Tanabe A Hirabayashi K Frequencies of dendritic cells (myeloid DC and plasmacytoid DC) and their ratio reduced in pregnant women: comparison with umbilical cord blood and normal healthy adults. Hum Immunol (2003) 64(12):1144–51. 10.1016/j.humimm.2003.08.342 14630396

[26] Bachy V Williams DJ Ibrahim MA. Altered dendritic cell function in normal pregnancy. J Reprod Immunol (2008) 78(1):11–21. 10.1016/j.jri.2007.09.004 18006075

[27] Widner B Leblhuber F Fuchs D Increased neopterin production and tryptophan degradation in advanced Parkinson’s disease. J Neural Transm (Vienna) (2002) 109(2):181–9. 10.1007/s007020200014 12075858

[28] Huber C Batchelor JR Fuchs D Hausen A Lang A Niederwieser D Immune response-associated production of neopterin. Release from macrophages primarily under control of interferon-gamma. J Exp Med (1984) 160(1):310–6. 10.1084/jem.160.1.310 6429267 PMC2187425

[29] Tachibana D Shinataku H Fukumasu H Yamamasu S Fukumasu Y Iwanaga N Neopterin and biopterin levels in pregnancy, in chemistry and biology of pteridines and folates. In: Milstien S editors. Proceedings of the 12th International Symposium on Pteridines and Folates, National Institutes of Health; 2001 Jun 17–22; Bethesda, Maryland, Boston, MA, USA: Springer (2002). p. 387–91.

[30] Navolan DB Vladareanu S Lahdou I Ciohat I Kleist C Grigoras D Early pregnancy serum neopterin concentrations predict spontaneous preterm birth in asymptomatic pregnant women. J Perinat Med (2016) 44(5):517–22. 10.1515/jpm-2015-0081 25918916

[31] Szekeres-Bartho J Polgar B. PIBF: the double edged sword. Pregnancy and tumor. Am J Reprod Immunol (2010) 64(2):77–86. 10.1111/j.1600-0897.2010.00833.x 20367622

[32] Cohen RA Check JH Dougherty MP. Evidence that exposure to progesterone alone is a sufficient stimulus to cause a precipitous rise in the immunomodulatory protein the progesterone induced blocking factor (PIBF). J Assist Reprod Genet (2016) 33(2):221–9. 10.1007/s10815-015-0619-7 26634256 PMC4759003

[33] Laskarin G Tokmadzić VS Strbo N Bogović T Szekeres-Bartho J Randić L Progesterone induced blocking factor (PIBF) mediates progesterone induced suppression of decidual lymphocyte cytotoxicity. Am J Reprod Immunol (2002) 48(4):201–9. 10.1034/j.1600-0897.2002.01133.x 12516630

[34] Szekeres-Bartho J Barakonyi A Polgar B Par G Faust Z Palkovics T The role of gamma/delta T cells in progesterone-mediated immunomodulation during pregnancy: a review. Am J Reprod Immunol (1999) 42(1):44–8. 10.1111/j.1600-0897.1999.tb00464.x 10429766

[35] Johansson ED Plasma levels of progesterone in pregnancy measured by a rapid competitive protein binding technique. Acta Endocrinol (Copenh) (1969) 61(4):607–17. 5409083 10.1530/acta.0.0610607

[36] Truong HM Sim MS Dillon M Uittenbogaart CH Dickover R Plaeger SF Correlation of immune activation during late pregnancy and early postpartum with increases in plasma HIV RNA, CD4/CD8 T cells, and serum activation markers. Clin Vaccine Immunol (2010) 17(12):2024–8. 10.1128/CVI.00088-10 20980480 PMC3008186

[37] Tilburgs T Schonkeren D Eikmans M Nagtzaam NM Datema G Swings GM Human decidual tissue contains differentiated CD8+ effector-memory T cells with unique properties. J Immunol (2012) 185(7):4470–7. 10.4049/jimmunol.0903597 20817873

[38] Wegorzewska M Nijagal A Wong CM Le T Lescano N Tang Q Fetal intervention increases maternal T cell awareness of the foreign conceptus and can lead to immune-mediated fetal demise. J Immunol (2014) 192(4):1938–45. 10.4049/jimmunol.1302403 24415782 PMC4268439

[39] Imami N Pires A Hardy G Wilson J Gazzard B Gotch F. A balanced type 1/type 2 response is associated with long-term nonprogressive human immunodeficiency virus type 1 infection. J Virol (2002) 76(18):9011–23. 10.1128/JVI.76.18.9011-9023.2002 12186885 PMC136425

[40] Imami N Hardy GA Nelson MR Morris-Jones S Al-Shahi R Antonopoulos C Induction of HIV-1-specific T cell responses by administration of cytokines in late-stage patients receiving highly active anti-retroviral therapy. Clin Exp Immunol (1999) 118(1):78–86. 10.1046/j.1365-2249.1999.01012.x 10540163 PMC1905397

[41] Burton CT Gotch F Imami N. Rapid qualitative and quantitative analysis of T-cell responses in HIV-1-infected individuals receiving successful HAART and HIV-1 sero-negative controls: concomitant assessment of perforin, IFN-gamma and IL-4 secretion. J Immunol Methods (2006) 308(1–2):216–30. 10.1016/j.jim.2005.11.005 16388819

[42] Widner B Werner ER Schennach H Wachter H Fuchs D Simultaneous measurement of serum tryptophan and kynurenine by HPLC. Clin Chem (1997) 43(12):2424–6. 9439467

[43] Holmes VA Wallace JM Gilmore WS McFaul P Alexander HD. Plasma levels of the immunomodulatory cytokine interleukin-10 during normal human pregnancy: a longitudinal study. Cytokine (2003) 21(6):265–9. 10.1016/S1043-4666(03)00097-8 12823999

[44] Curry AE Vogel I Skogstrand K Drews C Schendel DE Flanders WD Maternal plasma cytokines in early- and mid-gestation of normal human pregnancy and their association with maternal factors. J Reprod Immunol (2008) 77(2):152–60. 10.1016/j.jri.2007.06.051 17692390

[45] Denney JM Nelson EL Wadhwa PD Waters TP Mathew L Chung EK Longitudinal modulation of immune system cytokine profile during pregnancy. Cytokine (2011) 53(2):170–7. 10.1016/j.cyto.2010.11.005 21123081 PMC4610033

[46] Kronborg CS Gjedsted J Vittinghus E Hansen TK Allen J Knudsen UB. Longitudinal measurement of cytokines in pre-eclamptic and normotensive pregnancies. Acta Obstet Gynecol Scand (2011) 90(7):791–6. 10.1111/j.1600-0412.2011.01134.x 21595635

[47] Sallusto F Lenig D Förster R Lipp M Lanzavecchia A. Two subsets of memory T lymphocytes with distinct homing potentials and effector functions. Nature (1999) 401(6754):708–12. 10.1038/44385 10537110

[48] Amoudruz P Minang JT Sundström Y Nilsson C Lilja G Troye-Blomberg M Pregnancy, but not the allergic status, influences spontaneous and induced interleukin-1beta (IL-1beta), IL-6, IL-10 and IL-12 responses. Immunology (2006) 119(1):18–26. 10.1111/j.1365-2567.2006.02400.x 16764689 PMC1782335

[49] Gustafsson C Hummerdal P Matthiesen L Berg G Ekerfelt C Ernerudh J. Cytokine secretion in decidual mononuclear cells from term human pregnancy with or without labour: ELISPOT detection of IFN-gamma, IL-4, IL-10, TGF-beta and TNF-alpha. J Reprod Immunol (2006) 71(1):41–56. 10.1016/j.jri.2005.12.009 16730071

[50] Malavige GN Jeewandara C Alles KM Salimi M Gomes L Kamaladasa A Suppression of virus specific immune responses by IL-10 in acute dengue infection. PLoS Negl Trop Dis (2013) 7(9):e2409. 10.1371/journal.pntd.0002409 24040431 PMC3764236

[51] Wang L Pino-Lagos K de Vries VC Guleria I Sayegh MH Noelle RJ. Programmed death 1 ligand signaling regulates the generation of adaptive Foxp3+CD4+ regulatory T cells. Proc Natl Acad Sci U S A (2008) 105(27):9331–6. 10.1073/pnas.0710441105 18599457 PMC2442817

[52] D’Addio F Riella LV Mfarrej BG Chabtini L Adams LT Yeung M The link between the PDL1 costimulatory pathway and Th17 in fetomaternal tolerance. J Immunol (2011) 187(9):4530–41. 10.4049/jimmunol.1002031 21949023 PMC3197965

[53] Habicht A Dada S Jurewicz M Fife BT Yagita H Azuma M A link between PDL1 and T regulatory cells in fetomaternal tolerance. J Immunol (2007) 179(8):5211–9. 10.4049/jimmunol.179.8.5211 17911606

[54] Toldi G Saito S Shima T Halmos A Veresh Z Vásárhelyi B The frequency of peripheral blood CD4+ CD25high FoxP3+ and CD4+ CD25- FoxP3+ regulatory T cells in normal pregnancy and pre-eclampsia. Am J Reprod Immunol (2012) 68(2):175–80. 10.1111/j.1600-0897.2012.01145.x 22510013

[55] Schumacher A Costa SD Zenclussen AC. Endocrine factors modulating immune responses in pregnancy. Front Immunol (2014) 5:196. 10.3389/fimmu.2014.00196 24847324 PMC4021116

[56] Arck P Hansen PJ Mulac Jericevic B Piccinni MP Szekeres-Bartho J. Progesterone during pregnancy: endocrine-immune cross talk in mammalian species and the role of stress. Am J Reprod Immunol (2007) 58(3):268–79. 10.1111/j.1600-0897.2007.00512.x 17681043

[57] Check JH Dix E Sansoucie L. Support for the hypothesis that successful immunotherapy of various cancers can be achieved by inhibiting a progesterone associated immunomodulatory protein. Med Hypotheses (2009) 72(1):87–90. 10.1016/j.mehy.2008.05.042 18842348

[58] Schatz F Kayisli UA Vatandaslar E Ocak N Guller S Abrahams VM Toll-like receptor 4 expression in decidual cells and interstitial trophoblasts across human pregnancy. Am J Reprod Immunol (2012) 68:146–53. 10.1111/j.1600-0897.2012.01148.x 22564191 PMC3395732

[59] Stjernholm YV Progesterone in human pregnancy and parturition. In: Dubey PR, editor. Sex Hormones. Croatia: InTech (2012). p. 100–14. 10.5772/27055

[60] Martin N Höftmann T Politt E Hoppen HO Sohr M Günzel-Apel AR Morphological examination of the corpora lutea from pregnant bitches treated with different abortifacient regimes. Reprod Domest Anim (2009) 44(Suppl 2):185–9. 10.1111/j.1439-0531.2009.01430.x 19754564

[61] Grasso G Muscettola M The influence of beta-estradiol and progesterone on interferon gamma production in vitro. Int J Neurosci (1990) 51(3–4):315–7. 10.3109/00207459008999730 2126259

[62] Pettersson A Ciumas C Chirsky V Link H Huang YM Xiao BG. Dendritic cells exposed to estrogen in vitro exhibit therapeutic effects in ongoing experimental allergic encephalomyelitis. J Neuroimmunol (2004) 156(1–2):58–65. 10.1016/j.jneuroim.2004.07.004 15465596

[63] Xiao BG Liu X Link H. Antigen-specific T cell functions are suppressed over the estrogen-dendritic cell-indoleamine 2,3-dioxygenase axis. Steroids (2004) 69(10):653–9. 10.1016/j.steroids.2004.05.019 15465110

[64] Engler JB Kursawe N Solano ME Patas K Wehrmann S Heckmann N Glucocorticoid receptor in T cells mediates protection from autoimmunity in pregnancy. Proc Natl Acad Sci U S A (2017) 114(2):E181–90. 10.1073/pnas.1617115114 28049829 PMC5240705

[65] Kissick HT Sanda MG Dunn LK Pellegrini KL On ST Noel JK Androgens alter T-cell immunity by inhibiting T-helper 1 differentiation. Proc Natl Acad Sci U S A (2014) 111(27):9887–92. 10.1073/pnas.1402468111 24958858 PMC4103356

[66] Walecki M Eisel F Klug J Baal N Paradowska-Dogan A Wahle E Androgen receptor modulates Foxp3 expression in CD4+CD25+Foxp3+ regulatory T-cells. Mol Biol Cell (2015) 26(15):2845–57. 10.1091/mbc.E14-08-1323 26063731 PMC4571343

[67] Lissauer D Eldershaw SA Inman CF Coomarasamy A Moss PA Kilby MD. Progesterone promotes maternal-fetal tolerance by reducing human maternal T-cell polyfunctionality and inducing a specific cytokine profile. Eur J Immunol (2015) 45(10):2858–72. 10.1002/eji.201445404 26249148 PMC4833190

[68] Faust Z Laskarin G Rukavina D Szekeres-Bartho J. Progesterone-induced blocking factor inhibits degranulation of natural killer cells. Am J Reprod Immunol (1999) 42(2):71–5. 10476687

[69] Szekeres-Bartho J Autran B Debre P Andreu G Denver L Chaouat G. Immunoregulatory effects of a suppressor factor from healthy pregnant women’s lymphocytes after progesterone induction. Cell Immunol (1989) 122(2):281–94. 10.1016/0008-8749(89)90077-4 2527616

[70] Srivastava MD Thomas A Srivastava BI Check JH. Expression and modulation of progesterone induced blocking factor (PIBF) and innate immune factors in human leukemia cell lines by progesterone and mifepristone. Leuk Lymphoma (2007) 48(8):1610–7. 10.1080/10428190701471999 17701593

[71] Ivanova-Todorova E Kyurkchiev DS Nalbanski A Timeva T Shterev A Kyurkchiev SD Production and characterization of a novel monoclonal antibody against progesterone-induced blocking factor (PIBF). J Reprod Immunol (2008) 78(2):94–101. 10.1016/j.jri.2007.12.001 18243332

[72] Kozma N Halasz M Palkovics T Szekeres-Bartho J. The progesterone-induced blocking factor modulates the balance of PKC and intracellular Ca. Am J Reprod Immunol (2006) 55(2):122–9. 10.1111/j.1600-0897.2005.00337.x 16433831

[73] Vantourout P Hayday A Six-of-the-best: unique contributions of gammadelta T cells to immunology. Nat Rev Immunol (2013) 13(2):88–100. 10.1038/nri3384 23348415 PMC3951794

[74] Polgár B Nagy E Mikó E Varga P Szekeres-Barthó J. Urinary progesterone-induced blocking factor concentration is related to pregnancy outcome. Biol Reprod (2004) 71(5):1699–705. 10.1095/biolreprod.104.030437 15269099

[75] Tulchinsky D Hobel CJ Yeager E Marshall JR. Plasma estrone, estradiol, estriol, progesterone, and 17-hydroxyprogesterone in human pregnancy. I. Normal pregnancy. Am J Obstet Gynecol (1972) 112(8):1095–100. 10.1016/0002-9378(72)90185-8 5025870

[76] Xu Y He H Li C Shi Y Wang Q Li W Immunosuppressive effect of progesterone on dendritic cells in mice. J Reprod Immunol (2011) 91(1–2):17–23. 10.1016/j.jri.2011.06.101 21856019

[77] Martínez FF Knubel CP Sánchez MC Cervi L Motrán CC. Pregnancy-specific glycoprotein 1a activates dendritic cells to provide signals for Th17-, Th2-, and Treg-cell polarization. Eur J Immunol (2012) 42(6):1573–84. 10.1002/eji.201142140 22678910

[78] Schröcksnadel K Widner B Bergant A Neurauter G Schennach H Schröcksnadel H Longitudinal study of tryptophan degradation during and after pregnancy. Life Sci (2003) 72(7):785–93. 10.1016/S0024-3205(02)02304-4 12479977

[79] Curti A Trabanelli S Salvestrini V Baccarani M Lemoli RM. The role of indoleamine 2,3-dioxygenase in the induction of immune tolerance: focus on hematology. Blood (2009) 113(11):2394–401. 10.1182/blood-2008-07-144485 19023117

[80] Tsuji A Nakata C Sano M Fukuwatari T Shibata K. L-tryptophan metabolism in pregnant mice fed a high L-tryptophan diet and the effect on maternal, placental, and fetal growth. Int J Tryptophan Res (2013) 6:21–33. 10.4137/IJTR.S12715 24009424 PMC3748091

[81] Mikyas Y Aziz N Harawa N Gorre M Neagos N Nogueira M Immunologic activation during pregnancy: serial measurement of lymphocyte phenotype and serum activation molecules in HIV-infected and uninfected women. J Reprod Immunol (1997) 33(2):157–70. 10.1016/S0165-0378(97)00018-1 9234214

[82] Fuchs D Weiss G Reibnegger G Wachter H. The role of neopterin as a monitor of cellular immune activation in transplantation, inflammatory, infectious, and malignant diseases. Crit Rev Clin Lab Sci (1992) 29(3–4):307–41. 10.3109/10408369209114604 1489521

[83] Wirleitner B Reider D Ebner S Böck G Widner B Jaeger M Monocyte-derived dendritic cells release neopterin. J Leukoc Biol (2002) 72(6):1148–53. 12488496

[84] Nair RR Khanna A Singh K Association of FAS -1377 G>A and FAS -670 A>G functional polymorphisms of FAS gene of cell death pathway with recurrent early pregnancy loss risk. J Reprod Immunol (2012) 93(2):114–8. 10.1016/j.jri.2011.12.004 22386066

[85] Thellin O Coumans B Zorzi W Igout A Heinen E. Tolerance to the foeto-placental ‘graft’: ten ways to support a child for nine months. Curr Opin Immunol (2000) 12(6):731–7. 10.1016/S0952-7915(00)00170-9 11102780

[86] Vacchio MS Hodes RJ. Fetal expression of Fas ligand is necessary and sufficient for induction of CD8 T cell tolerance to the fetal antigen H-Y during pregnancy. J Immunol (2005) 174(8):4657–61. 10.4049/jimmunol.174.8.4657 15814689

[87] Paulsen M Valentin S Mathew B Adam-Klages S Bertsch U Lavrik I Modulation of CD4+ T-cell activation by CD95 co-stimulation. Cell Death Differ (2011) 18(4):619–31. 10.1038/cdd.2010.134 21052094 PMC3131913

[88] Zhu C Anderson AC Schubart A Xiong H Imitola J Khoury SJ The Tim-3 ligand galectin-9 negatively regulates T helper type 1 immunity. Nat Immunol (2005) 6(12):1245–52. 10.1038/ni1271 16286920

[89] Anderson AC Anderson DE. TIM-3 in autoimmunity. Curr Opin Immunol (2006) 18(6):665–9. 10.1016/j.coi.2006.09.009 17011764

[90] Zhao J Lei Z Liu Y Li B Zhang L Fang H Human pregnancy up-regulates Tim-3 in innate immune cells for systemic immunity. J Immunol (2009) 182(10):6618–24. 10.4049/jimmunol.0803876 19414817

[91] Vassiliadis S Ranella A Papadimitriou L Makrygiannakis A Athanassakis I Serum levels of pro- and anti-inflammatory cytokines in non-pregnant women, during pregnancy, labour and abortion. Mediators Inflamm (1998) 7(2):69–72. 10.1080/09629359891199 9836491 PMC1781827

[92] Kruse N Greif M Moriabadi NF Marx L Toyka KV Rieckmann P. Variations in cytokine mRNA expression during normal human pregnancy. Clin Exp Immunol (2000) 119(2):317–22. 10.1046/j.1365-2249.2000.01123.x 10632669 PMC1905505

[93] Goulopoulou S Matsumoto T Bomfim GF Webb RC. Toll-like receptor 9 activation: a novel mechanism linking placenta-derived mitochondrial DNA and vascular dysfunction in pre-eclampsia. Clin Sci (Lond) (2012) 123(7):429–35. 10.1042/CS20120130 22671429 PMC4004352

[94] Reddy A Zhong XY Rusterholz C Hahn S Holzgreve W Redman CW The effect of labour and placental separation on the shedding of syncytiotrophoblast microparticles, cell-free DNA and mRNA in normal pregnancy and pre-eclampsia. Placenta (2008) 29(11):942–9. 10.1016/j.placenta.2008.08.018 18834630

[95] Joerger-Messerli MS Hoesli IM Rusterholz C Lapaire O. Stimulation of monocytes by placental microparticles involves toll-like receptors and nuclear factor kappa-light-chain-enhancer of activated B cells. Front Immunol (2014) 5:173. 10.3389/fimmu.2014.00173 24782870 PMC3995043

[96] Nancy P Erlebacher A. T cell behavior at the maternal-fetal interface. Int J Dev Biol (2014) 58(2–4):189–98. 10.1387/ijdb.140054ae 25023685 PMC4212519

[97] Norton MT Fortner KA Oppenheimer KH Bonney EA. Evidence that CD8 T-cell homeostasis and function remain intact during murine pregnancy. Immunology (2010) 131(3):426–37. 10.1111/j.1365-2567.2010.03316.x 20553337 PMC2996563

[98] Erlebacher A Vencato D Price KA Zhang D Glimcher LH. Constraints in antigen presentation severely restrict T cell recognition of the allogeneic fetus. J Clin Invest (2007) 117(5):1399–411. 10.1172/JCI28214 17446933 PMC1849983

