# Supplementary material for: Changes in T Cell and Dendritic Cell Phenotype from Mid to Late Pregnancy Are Indicative of a Shift from Immune Tolerance to Immune Activation
Source: Front Immunol. 2017 Sep 15;8:1138. doi: 10.3389/fimmu.2017.01138 (PMC5605754; doi:10.3389/fimmu.2017.01138)
Supplement: Supplementary file 1 [file image_1.pdf]

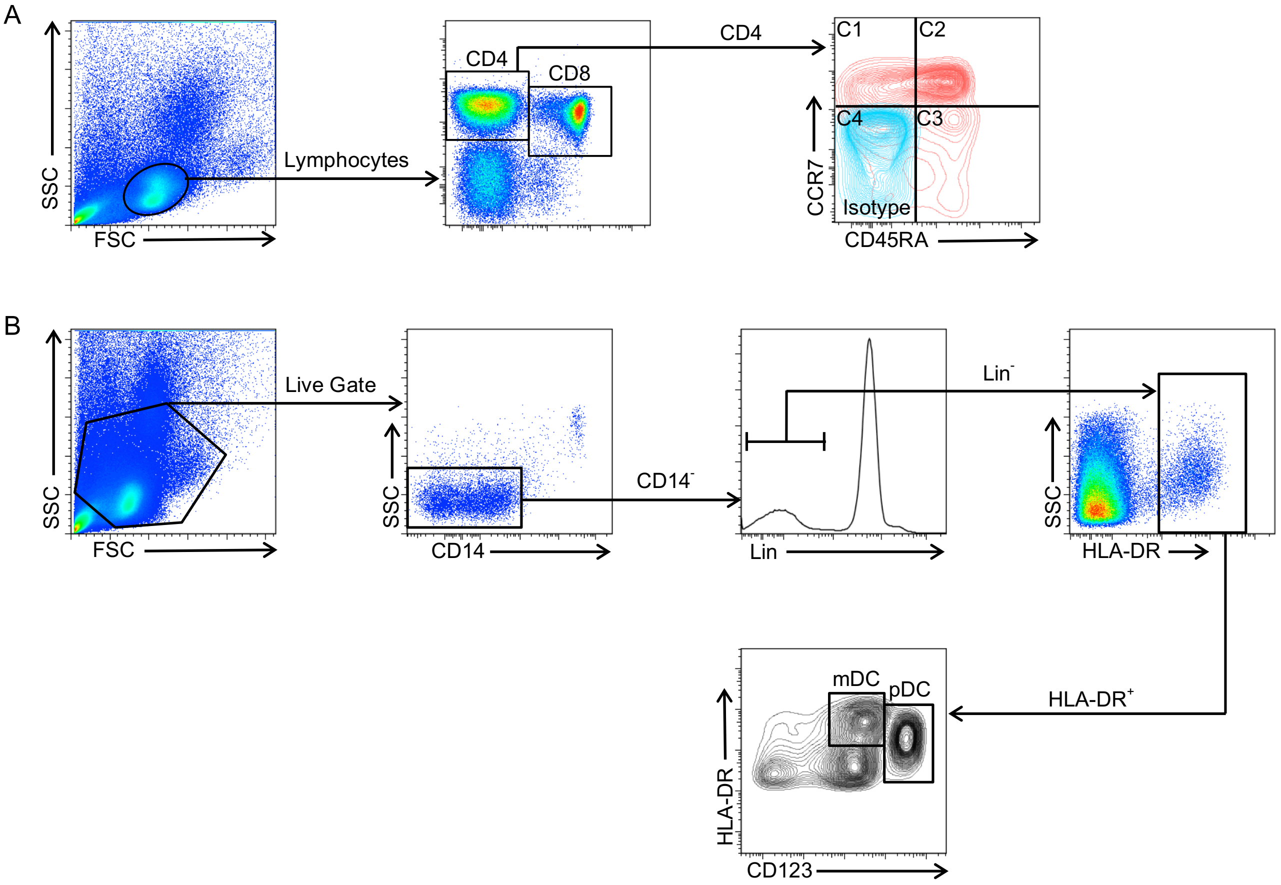

Supplemental Figure 1. (A) Flow cytometry plots showing the gating strategy used to identify T_{CM} (C1), naïve (C2), T_{TEMRA} (C3) and T_{EM} (C4) CD4 lymphocytes. (B) Flow cytometry plots and histograms showing the gating strategy used to identify mDC (CD14$^-$Lin$^-$HLA-DR^{hi}CD123$^-$) and pDC (CD14$^-$Lin$^-$HLA-DR$^+$CD123$^+$).
